# Analysis of MarketScan Data for Immunosuppressive Conditions and Hospitalizations for Acute Respiratory Illness, United States

**DOI:** 10.3201/eid2608.191493

**Published:** 2020-08

**Authors:** Manish Patel, Jufu Chen, Sara Kim, Shikha Garg, Brendan Flannery, Zaid Haddadin, Danielle Rankin, Natasha Halasa, H. Keipp Talbot, Carrie Reed

**Affiliations:** Centers for Disease Control and Prevention, Atlanta, Georgia, USA (M. Patel, J. Chen, S. Kim, S. Garg, B. Flannery, C. Reed);; Vanderbilt University, Nashville, Tennessee, USA (Z. Haddadin, D. Rankin, N. Halasa, H. Keipp Talbot)

**Keywords:** immunosuppressive conditions, biologics, cancer, influenza, respiratory infections, acute respiratory illness, vaccines, infections, hospitalizations, analysis, International Classification of Diseases, MarketScan, United States

## Abstract

Increasing use of immunosuppressive biologic therapies poses a challenge for infectious diseases. Immunosuppressed patients have a high risk for influenza complications and an impaired immune response to vaccines. The total burden of immunosuppressive conditions in the United States, including those receiving emerging biologic therapies, remains unknown. We used the national claims database MarketScan to estimate the prevalence of immunosuppressive conditions and risk for acute respiratory illnesses (ARIs). We studied 47.2 million unique enrollees, representing 115 million person-years of observation during 2012–2017, and identified immunosuppressive conditions in 6.2% adults 18–64 years of age and 2.6% of children <18 years of age. Among 542,105 ARI hospitalizations, 32% of patients had immunosuppressive conditions. The risk for ARI hospitalizations was higher among enrollees with immunosuppression than among nonimmunosuppressed enrollees. Future efforts should focus on developing improved strategies, including vaccines, for preventing influenza in immunosuppressed patients, who are an increasing population in the United States.

Influenza is a common cause of illness and death in the United States and affects persons of all ages (*1*). Risk for complications from infection is higher in subpopulations, such as persons with immunosuppressive conditions (*2*,*3*). In recent years, an increasing number of patients are receiving biologic or immune-modulating agents with immunosuppressive potential (*4*,*5*). Although data exist on the prevalence of some immunosuppressive conditions, the total burden of these conditions in the United States remains unknown, particularly when considering patients who are receiving emerging immunosuppressive therapies (*6*–*8*).

Influenza vaccination prevents disease and averts severe outcomes, such as hospitalization and death (*1*,*9*). A meta-analysis of observational studies of influenza vaccines identified that pooled vaccine effectiveness was 33%–67% against medically attended, laboratory-confirmed influenza illness in the overall population (*10*). However, a review of immunogenicity studies suggests that antibody responses to inactivated influenza vaccines (IIVs) in persons who are immunocompromised could be suboptimal compared with persons without immunosuppression (*11*).

Clinical effectiveness data are sparse, but a recent observational study demonstrated lower vaccine effectiveness against influenza illness (≈20%) in patients with cancer compared with the general population (≈42%) (*12*,*13*). Increasing efficacy of influenza vaccines in immunosuppressed populations might substantially improve population benefits of influenza vaccines. Establishing a case definition for and quantifying the burden of immunosuppressive conditions might facilitate evaluation and use of influenza vaccines to enhance immune response in this high-risk target group.

IIVs that contain egg-propagated vaccine viruses and a standard dose of 15 μg of hemagglutinin antigen of each virus per dose, without adjuvant, are the most commonly used vaccines worldwide (*14*). In recent years, 2 enhanced IIVs, MF59-adjuvanted standard-dose IIV and a high-dose IIV that contains 4 times the hemagglutinin antigen of each virus compared with the standard-dose IIV, have been developed to improve the immune responses to and efficacy of standard-dose IIVs (*15*,*16*). Both vaccines are currently licensed in the United States for use in older adults (*9*). High-dose IIV has also met prespecified criteria for superior efficacy against laboratory-confirmed influenza compared with standard-dose IIV (*15*,*17*). Although these enhanced IIVs are not yet licensed for use in US patients <65 years of age, some evidence suggests that humoral immune responses to these vaccines might also be greater than responses to standard IIVs in adults 18–64 years of age who have immunosuppressive conditions (*18*,*19*).

In this study, we created and used case definitions for immunosuppressive conditions by using a modified version of an algorithm implemented by previous investigators (*20*). Our primary objective was to determine the prevalence of immunosuppressive conditions in the US population among MarketScan (Truven Health MarketScan, https://marketscan.truvenhealth.com) enrollees <65 years of age. We recognized that International Classification of Diseases (ICD) and drug codes might not accurately capture enrollees with impaired immune systems. Thus, our secondary objective was to explore whether rates of influenza vaccination and hospitalization for acute respiratory infection (ARI) differed between those with and without immunosuppressive conditions identified by our case definitions.

## Methods

### Data Sources

We analyzed the MarketScan Commercial Claims and Medicare data from August 1, 2012, through July 31, 2017, to explore the prevalence of immunosuppressive conditions. We calculated rates of ARI hospitalizations among these enrollees relative to enrollees without immunosuppressive conditions. MarketScan is a de-identified commercial insurance claims database representing 30–50 million persons per year from >160 large employers and health plans representing all 50 US states (*21*). The Medicare database includes Medicare-eligible retirees with employer-sponsored Medicare Supplemental plans. The database includes healthcare claims with diagnosis and procedure codes for medical encounters and all outpatient prescription medications. Variables we examined included age, sex, influenza vaccination, and medications, as well as codes from the ICD, 9th Revision, Clinical Modification (ICD-9-CM), or ICD, 10th Revision, Clinical Modification (ICD-10-CM), for immunosuppressive conditions (any medical encounter/claim) and hospitalizations for pneumonia, influenza, and diseases of the respiratory system. We restricted our sample to those enrolled and covered by the drug benefit program during the study years.

### Immunosuppressive Conditions

**Figure 1:**
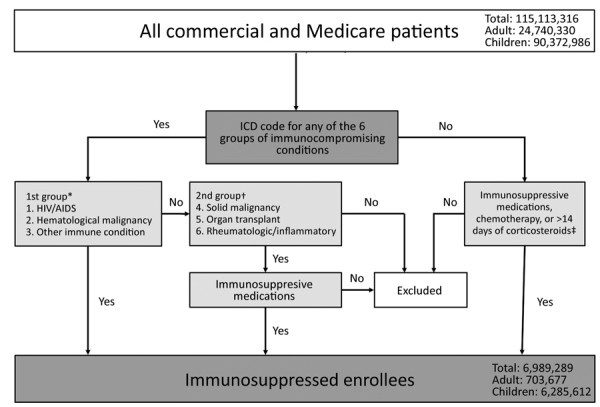
Algorithm for case definitions of immunosuppressive conditions in MarketScan claims database of Commercial and Medicare enrollees, United States, August 2012–July 2017. *These 3 conditions were deemed to be immunosuppressive. †These 3 conditions were deemed to be immunosuppressive only if enrollees were given chemotherapeutic agents or immune-modulating agents or if enrollees who had rheumatologic or inflammatory conditions were receiving systemic corticosteroids. ‡We deemed that enrollees might be given chemotherapeutic agents or immune-modulating agents and not be captured by ICD codes (from the 9th Revision, Clinical Modification, or 10th Revision, Clinical Modification) for the 6 potential immunosuppressive conditions described in the first 2 footnotes. We excluded treatment with corticosteroids for <14 days from these groups to avoid capturing enrollees who might be receiving short-term bursts of corticosteroids (e.g., those with asthma). ICD, International Classification of Diseases.

Greenberg et al. have previously established an algorithm for identifying patients with active immunosuppression on the basis of ICD and Current Procedural Terminology (CPT) codes in a large database of patients who were acutely ill with sepsis (*20*). We slightly modified the approach by Greenberg et al. to derive a case definition of immunosuppressive conditions based on 6 groups of diseases and 3 classes of medications (Figure 1). The Infection Diseases Society of America has published detailed guidance for the selection and timing of vaccines for persons with specific immunocompromising conditions but does not consider specific ICD codes (*5*). We reviewed those guidelines to identify additional immunocompromising conditions not included in the Greenberg algorithm (sickle cell disease, asplenia, and psoriatic arthritis) and assessed whether inclusion of these conditions would affect our results.

We considered 3 groups of enrollees to be immunosuppressed: 1) persons with symptomatic HIV/AIDS (excluding asymptomatic HIV), hematologic malignancy, or other intrinsic immune conditions; 2) persons with solid malignancy, organ transplant, rheumatologic, or other inflammatory conditions that were deemed immunosuppressed if patients received chemotherapy or an immune modulator; or rheumatologic or other inflammatory conditions who received systemic (nontopical, noninhaled) steroids; 3) any enrollee not in the first 2 groups who received chemotherapy, an immune modulator, or systemic steroids for >14 days (not considered by Greenberg et al.). Enrollees receiving corticosteroids for <14 days were not considered immunosuppressed because most probably were receiving short-term burst doses, which has low immunosuppressive potential (*5*). The 3 enrollee groups were mutually exclusive. Enrollees who did not meet the immunosuppressed case definition were considered nonimmunosuppressed.

We examined data for persons of all ages who had continuous enrollment in 1 insurance plan during 2 consecutive years. We used ICD-9 and ICD-10 codes from any medical encounter/claim to identify immunosuppressive conditions during the 12-month enrollment periods, including influenza seasons from August 1, 2012, through July 31, 2017. Enrollees were considered immunosuppressed during the enrollment year if they had >1 hospitalization or 2 separate outpatient visits listing a corresponding ICD-9 code before October 1, 2015 or ICD-10 code after October 1, 2015 (Table 1), or were prescribed 1 of the listed medications during each of the 12 months (August 1–July 31) of the study period (Table 2). For the purposes of analysis, we considered these persons immunosuppressed for that entire 12-month period.

**Table 1:** Conditions and ICD-9-CM and ICD-10-CM codes used to identify enrollees with immunosuppression in MarketScan database, United States, July 2012–August 2017*

Condition	ICD-9 codes	ICD-10 codes	
HIV/AIDS†			
HIV/AIDS disease	042	B20-B24	
Hematologic malignancy			
Lymphatic and hematopoietic tissue malignancy	200–208	C81-C83; C88-C96	
Other immune conditions‡			
Disorders of immune mechanism	279	D89	
Neutropenia	288.0	D70	
Functional disorders of neutrophils	288.1	D71	
Genetic anomalies of leukocytes	288.2	D72.0	
Decreased leukocyte count	288.5	D72.81	
Leukocyte disease NEC	288.8	D72.89	
Leukocyte disease NOS	288.9	D72.9	
Myelofibrosis	289.83	D75.81	
Blood diseases NEC	289.89	D47.4; D75.89; D75.9; D89.2	
Blood diseases NOS	289.9	D75.9; D75.89	
Immunologic findings NEC	795.7	R76; R83.4-R87.4; R89.4	
Nonspecific immune findings NEC and NOS	795.79	R76; R83.4-R87.4; R89.4	
Solid malignancy			
Organ/system malignant tumors	140–199	C00-C07; C11-C19; C22-C80; Z85	
Neuroendocrine tumors	209	C7A; C7B; D3A	
Neoplasms of uncertain behavior	235–239	D00-D49	
Organ transplant§			
Complications of transplanted organ	996.8	T86	
Organ transplant status	V42	Z94; Z98.85	
Rheumatologic/inflammatory¶			
Sarcoidosis	135	D86	
Amyloidosis NOS	277.3	E85	
Familial Mediterranean fever	277.31	E85.0; M04	
Amyloidosis NEC	277.39	E85.1; E85.3; E85.8	
Multiple sclerosis	340	G35	
Other CNS demyelination	341	G36; G37.1; G37.3; G37.8; G37.9	
Acute infective polyneuritis	357	G61.0; G61.9	
Acute myocarditis	422	I40	
Polyarteritis nodosa and other	446	M30	
Allergic alveolitis/pneumonitis NOS	495.9	T78.40; J67.9	
Other alveolar pneumonopathy	516	J84.01; J84.02; J84.09	
Enteritis and colitis	555–558	K50-K52	
Lupus erythematosus	695.4	L93.0; L93.2; M32	
Diffuse connective tissue disease	710	L94; M35.8; M35.9	
Arthropathy with infection	711	M12.9; M01.X0; M02.10	
Crystal arthropathies	712	M11	
Rheumatoid arthritis/inflammatory polyarthropathy	714	M05-M14	
Inflammatory spondylopathies	720	M46	
Polymyalgia rheumatica	725	M31.5; M35.3	

*CNS, central nervous system; ICD9-CM, International Classification of Diseases, 9th Revision, Clinical Modification; ICD-10-CM, International Classification of Diseases, 10th Revision, Clinical Modification; NOS, not otherwise specified; NEC, necrotizing enterocolitis. †Excludes asymptomatic HIV codes of ICD-9 (V08) and ICD-10 (Z21). ‡Sickle cell disease, asplenia, and psoriatic arthritis were not included in the Greenberg algorithm (*20*) but are considered to have immune deficiencies by Infectious Diseases Society of America guidelines (*5*). Adding these to the algorithm only increased the prevalence of immunosuppressive conditions by 0.1%. §Bone marrow and peripheral stem cell transplant were considered under organ transplant and only considered immunosuppressed if enrollees were currently being given chemotherapeutic agents or immune modulators. Considering these enrollees under other immune conditions in which immunosuppressed does not require receipt of chemotherapeutic agents or immune modulators would increase the overall prevalence of immunosuppressed by 0.01%. ¶Psoriatic arthritis was not included in the Greenberg algorithm and could be an indication for immunosuppressive treatment. Adding this condition did not increase the prevalence of immunosuppressive conditions.

**Table 2:** Immunosuppressive agents used in analysis of MarketScan data for immunosuppressive conditions and hospitalizations for acute respiratory illness, United States

Agent
Chemotherapeutic
Aldesleukin
Alemtuzumab
Altretamine
Amifostine
Arsenic trioxide
Asparaginase
Azacitidine
Bendamustine hydrochloride
Bevacizumab
Bexarotene
Bortezomib
Brentuximab vedotin
Busulfan
Cabazitaxel
Capecitabine
Carboplatin
Carfilzomib
Carmustine
Cetuximab
Chlorambucil
Cisplatin
Cladribine
Clofarabine
Cyclophosphamide
Dacarbazine
Dactinomycin
Dasatinib
Daunorubicin citrate liposome
Decitabine
Denileukin diftitox
Docetaxel
Etoposide
Everolimus
Floxuridine
Fluorouracil
Gefitinib
Ifosfamide
Ipilimumab
Ixabepilone
Lomustine
Melphalan
Mercaptopurine
Mesna
Methotrexate
Mitomycin
Mitotane
Nelarabine
Ofatumumab
Oxaliplatin
Paclitaxel
Panitumumab
Pegaspargase
Pemetrexed
Pentostatin
Pertuzumab
Plicamycin
Pralatrexate
Rituximab
Romidepsin
Streptozocin
Temozolomide
Teniposide
Thioguanine
Thiotepa
Trastuzumab
Tretinoin
Vorinostat
Immune-modulating
Abatacept
Adalimumab
Alefacept
Anakinra
Auranofin
Aurothioglucose
Azathioprine
Basiliximab
Belatacept
Belimumab
Certolizumab pegol
Cyclosporine
Daclizumab
Denosumab
Eculizumab
Efalizumab
Etanercept
Gold sodium thiomalate
Golimumab
Infliximab
Interferon alfacon-1
Leflunomide
Lenalidomide
Mycophenolate mofetil
Natalizumab
Palifermin
Palivizumab
Pegademase bovine
Pimecrolimus
Sirolimus
Tacrolimus
Thalidomide
Tocilizumab
Ustekinumab
Systemic corticosteroids
Dexamethasone
Methylprednisolone
Prednisolone
Prednisone

### Acute Respiratory Illness Hospitalizations

All ICD codes that we used for immunosuppressive conditions might not necessarily be specific for conditions that impair immunity. Thus, we also evaluated risk for ARI hospitalization among patients who had immunosuppressive conditions in the MarketScan population. We identified ARI hospitalizations for pneumonia, influenza, and diseases of the respiratory system based on the first 3 discharge diagnosis ICD-9 or ICD-10 codes during August 1–July 31 in the 5 study years. Codes included 460–466 and 480–488 before October 1, 2015 (ICD-9-CM), and J00–J06, J09–J18, and J20–J22 after October 1, 2015 (ICD-10-CM). Data are limited on the validity of these ARI hospitalization codes overall and their position on the discharge diagnosis (*22*). Using codes in any position improves sensitivity but decreases positive predictive value. To balance sensitivity and specificity, we restricted discharge diagnoses to the first 3 positions and assumed that relative risk for ARI hospitalization between immunosuppressed and nonimmunosuppressed enrollees based on these codes would be unaffected. We inferred that higher relative rates of ARI hospitalizations among immunosuppressed enrollees would support the notion that the cohort of patients identified by our case definitions had some degree of immunosuppression overall.

### Influenza Vaccination

We identified enrollees who received influenza vaccine by using CPT codes (Table 3). We assumed that the relative adjusted vaccination rates between immunosuppressed and nonimmunosuppressed populations would reflect differences in influenza vaccine uptake.

**Table 3:** Codes for influenza vaccine used in analysis of MarketScan data for immunosuppressive conditions and hospitalizations for acute respiratory illness, United States

CPT no.*	Vaccine type
90653	Influenza virus vaccine, inactivated, subunit, adjuvanted, for intramuscular use
90654	influenza, seasonal, intradermal, preservative free
90655	Influenza virus vaccine, split virus, no preservative, for children 6–35 mo of age, for intramuscular use
90656	Influenza virus vaccine, split virus, no preservative, for use in persons >3 y of age, for intramuscular use
90657	Influenza virus vaccine, split virus, for children 6–35 mo of age, for intramuscular use
90658	Influenza virus vaccine, split virus, for use in persons >3 y of age, for intramuscular use
90659	Influenza, whole
90660	Influenza virus vaccine, live, for intranasal use
90661	Influenza virus vaccine, derived from cell cultures, subunit, preservative and antimicrobial drug free, for intramuscular use
90662	Influenza, high dose seasonal
90663	Influenza virus vaccine, pandemic H1N1
90664	Influenza virus vaccine, pandemic formulation, live, for intranasal use
90666	Influenza virus vaccine, pandemic formulation, split virus, preservative free, for intramuscular use
90667	Influenza virus vaccine, pandemic formulation, split virus, adjuvanted, for intramuscular use
90668	Influenza virus vaccine, pandemic formulation, split virus, for intramuscular use
90724	Influenza, unspecified formulation
90470	H1N1 immunization administration (intramuscular, intranasal)
90672	Influenza virus vaccine, quadrivalent, live, for intranasal use
90673	Influenza virus vaccine, trivalent, derived from recombinant DNA (recombinant influenza vaccine 3), hemagglutnin protein only, preservative and antimicrobial drug free, for intramuscular use
90685	Influenza virus vaccine, quadrivalent, split virus, preservative free, when administered to children 6–35 mo of age, for intramuscular use
90686	Influenza virus vaccine, quadrivalent, split virus, preservative free, when administered to children >3 y of age, for intramuscular use
90687	Influenza virus vaccine, quadrivalent, when administered to children 6–35 mo of age, for intramuscular use (not recognized by Medicare)
90688	Influenza virus vaccine, quadrivalent, when administered to persons >3 y, for intramuscular use (not recognized by Medicare)

*CPT, Current Procedural Terminology.

### Statistical Analysis

We examined prevalence of immunosuppressive conditions among all enrollees during each influenza season, stratified by age groups (0–8 years, 9–17 years, 18–49 years, and 50–64 years). We calculated relative incidence rates and 95% CIs of ARI hospitalization and influenza vaccination for enrollees with and without immunosuppressive conditions by using a generalized linear model with binomial distribution and log link function. We calculated 95% CIs for incidence rates based on the assumption that incidence rates followed a Poisson distribution. We compared rates for the entire year (August 1–July 31) and for December through March, the 4 months with the highest detection of influenza by surveillance data (*23*). We selected age, year, and sex, a priori, and adjusted the relative rates of ARI for these factors. We calculated person-time by using the total months each enrollee spent in a health plan supplying data to MarketScan during each study period. We conducted all analyses by using SAS version 9.4 (https://www.sas.com). The p values were 2-sided, and we considered a p value <0.05 as being statistically significant.

## Results

During August 2012–July 2017, a total of 47.2 million unique enrollees representing 115 million person-years of observation were included in the US MarketScan database (Table 4). Some enrollees did not complete an entire year of follow-up; 87% were enrolled for an entire year and 95% for >10 months. Age distribution of all enrollees compared with those with immunosuppressive conditions varied: 10% versus 2% for those <8 years of age, 12% versus 7% for those 9–17 years of age, 46% versus 38% for those 18–49 years of age, 25% versus 36% for those 50–64 years of age, and 8% versus 17% for those >65 years of age) (Table 5).

**Table 4:** Prevalence of immunosuppressive conditions by person age and sex for acute respiratory illness in the MarketScan database, United States, July 2012–August 2017

Characteristic	All enrollees, person-years, no. (%)*	Immunosuppressive conditions†	
		Person-years (%)	Prevalence/100 person-years, %
Total	115,113,322 (100)	6,823,509 (100)	5.9
Age, y			
<1–8	11,074,106 (10)	160,137 (2)	1.4
9–17	13,666,230 (12)	474,703 (7)	3.5
18–49	52,413,795 (46)	2,580,737 (38)	4.9
50–64	28,552,259 (25)	2,470,817 (36)	8.
>65	9,406,932 (8)	1,137,115 (17)	12.1
Sex			
M	55,282,285 (48)	2,597,852 (38)	4.7
F	59,831,037 (52)	4,225,657 (62)	7.1

*Person-time was calculated by using the total months each enrollee spent in a health plan supplying data to MarketScan during the study period. During August 2012–July 2017, a total of 47.2 million unique enrollees were in our MarketScan analysis. †See Figure 1. Denotes patients had International Classification of Diseases codes for either 3 immunosuppressive conditions OR 3 conditions + immunosuppressive pharmacologic treatment (chemotherapeutic agents or immune-modeling agents or systemic corticosteroids >14 d).

**Table 5:** Acute respiratory illness hospitalizations for patientsby age who had immunosuppressive conditions in MarketScan database, United States, July 2012–August 2017*

Characteristic	All ARI hospitalizations			ARI hospitalizations in immunosuppressed persons†		ARI in immunosuppressed versus nonimmunosuppressed persons, relative rate/1,000 person-years (95% CI)‡
	No.	Rate/1,000 person-years		No. (%)	Rate/1,000 person-years	
Year round, August–July						
Age, y						
All	542,105	4.7		173,665 (32.0)	25.5	4.2 (4.0–4.3)
>1–8	50,170	4.5		6,638 (13.2)	41.2	8.1 (7.8–8.4)
9–17	14,388	1.1		2,839 (19.7)	6.0	5.0 (4.7–5.4)
18–49	88,051	1.7		29,136 (33.1)	11.3	6.7 (6.5–6.9)
50–64	142,631	5.0		57,512 (40.3)	23.3	4.8 (4.6–4.9)
>65	246,865	26.2		77,540 31.4)	68.3	2.1 (2.0–2.1)
Peak influenza season, December–March						
Age, y						
All	240,856	6.3		77,308 (32.1)	34.0	4.3 (4.2–4.5)
<1–8	27,084	7.3		3,408 (12.6)	63.4	7.9 (7.5–8.3)
9–17	5,838	1.3		1,258 (21.5)	7.9	5.7 (5.1–6.4)
18–49	37,190	2.1		12,786 (34.4)	14.8	6.8 (6.8–7.4)
50–64	61,316	6.4		25,491 (41.6)	31.0	5.0 (4.8–5.2)
>65	109,428	34.9		34,365 (31.4)	90.8	2.1 (2.0–2.2)

*ARI, acute respiratory illness. †See Figure 1. Immunosuppressive denotes patients had International Classification of Diseases codes for either 3 immunosuppressive conditions OR 3 conditions plus immunosuppressive pharmacologic treatment (chemotherapeutic agents or immune modulators or systemic corticosteroids >14 d). ‡Relative rates of ARI were adjusted for year of hospitalization and sex.

**Figure 2:**
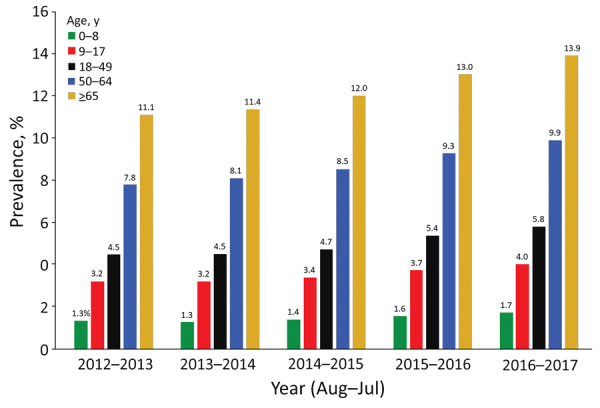
Prevalence of immunosuppressive conditions among children and adults in MarketScan claims database, United States, August 2012–July 2017.

Among 115 million person-years contributed during the study period, we found a prevalence of 5.9% for immunosuppressive conditions; prevalence was higher for female patients (7.1%) than for male patients (4.7%) (Table 5). Prevalence was 6.2% in the 18–64 year age group and 2.6% among children <18 years of age. Among enrollees with immunosuppressive conditions, 27% had HIV/AIDS, hematologic malignancy, or other intrinsic immune conditions; 36% had solid malignancies, organ transplant, or rheumatologic/inflammatory conditions treated with immunosuppressive medications; and 37% were related to immunosuppressive medications without the presence of an immunosuppressive medical conditions. We noted some increases in prevalence for immunosuppressive conditions during 2012–2017 for each of the age groups (Figure 2). When we included additional conditions not in the Greenberg algorithm (*20*) (sickle cell disease, asplenia, and psoriatic arthritis), our overall results did not change.

During the study period, we identified 542,105 ARI hospitalizations, of which 173,665 (32%) occurred in enrollees who had immunosuppression (Table 6). Annual rates of ARI were 4.2-fold higher among enrollees with immunosuppressive conditions (25.5 cases/1,000 person-years) compared with enrollees without immunosuppressive conditions (4.7 cases/1,000 person-years). When we restricted analysis to only the first 2 immunosuppressed groups without the immunosuppressive medications only group, we found that rates of ARI were 4.25-fold higher. Enrollees with immunosuppressive conditions accounted for 15% of the ARI hospitalizations among children <18 years of age and 38% of the ARI hospitalizations among persons 18–64 years of age.

**Table 6:** Age-specific influenza vaccination coverage and relative rates of coverage in enrollees with and without immunosuppressive conditions in MarketScan database, United States, July 2012–-August 2017

Age, y*	Vaccine coverage, %		Relative rate of vaccination, immunosuppressed versus nonimmunosuppressed, % (95% CI)
	Immunosuppressed	Nonimmunosuppressed	
>1–8	60.1	58.3	1.24 (1.23–1.25)
9–17	47.2	40.5	1.30 (1.29–1.30)
18–49	20.9	14.7	1.72 (1.71–1.73)
50–64	28.9	23.9	1.47 (1.46–1.48)
>65	30.3	27.9	1.27 (1.26–1.28)

*MarketScan vaccination data probably underestimate true influenza vaccine coverage in the population particularly for persons >65 years of age because not all vaccinations are billed to insurance companies (*24*). Relative rates of vaccination between immunosuppressed and nonimmunosuppressed should not be affected.

Age-stratified relative rates of ARI hospitalization adjusted for sex and year were higher among those with immunosuppressive conditions compared with immunocompetent enrollees <1–8 years of age (8.1%, 95% CI 7.8%–8.4%), 9–17 years of age (5.0%, 95% CI 4.7%–5.4%), 18–49 years of age (6.7%, 95% CI 6.5%–6.8%), and 50–64 years of age (4.8%, 95% CI 4.6%–4.9%) (Table 5). The relative rates of ARI hospitalization annually were similar to the relative rates during peak influenza months of December–March. Rates of influenza vaccination were also higher among enrollees 0–8 years of age (1.24-fold), 9–17 years of age (1.29-fold), 18–49 years of age (1.7-fold), and 50–64 years of age (1.4-fold) with immunosuppressive conditions compared with enrollees without immunosuppressive conditions (Table 5).

## Discussion

With the availability of new immunotherapy drugs and treatment practices for patients who have malignancies and chronic inflammatory diseases, persons with immunosuppressive conditions could account for an increasing proportion of patients in the United States (*4*,*25*–*28*). A systematic review suggests that these patients, especially those who have HIV, solid-organ and stem-cell transplants, and cancer, as well as patients receiving biologic agents, have an increased risk for influenza-related complications and suboptimal immune responses to standard IIV (*11*). Our analysis indicates that ≈6% of the enrollees in a large US claims database had immunosuppressive conditions during 2012–2017, which might represent some 12 million US persons if these rates are similar in the general US population (*29*). We found that risk for ARI hospitalization was 5–8-fold higher among enrollees <65 years of age who we classified as immunosuppressed, which is consistent with results of published studies that documented higher risk for complications from influenza and other pathogens in this patient population (*5*,*30*–*35*). The higher risk for ARI hospitalizations in enrollees with immunosuppression is also consistent with studies demonstrating inferior antibody responses to standard IIVs in immunosuppressed patients. (*11*). Some 38% of all patients 18–64 years of age hospitalized for ARI had immunosuppressive conditions. Our results indicate that immunosuppressed patient groups are disproportionally hospitalized for ARI and likely at high risk for complications from influenza.

Data are limited on whether enhanced vaccines would improve protection against influenza in immunosuppressed patients compared with standard IIVs. Immunosuppressive conditions are heterogeneous, comprising a wide range of immune states, some of which are time-variant, including receipt of immunosuppressive medications. Clinical trials of influenza vaccines typically include healthy persons. Studies assessing vaccine immunogenicity in patients with immunosuppressive conditions usually focus on a few specialized conditions; because of sample size limitations for efficacy endpoints, these studies typically focus on immunogenicity (*18*,*19*,*36*–*41*). A meta-analysis of studies has demonstrated strongly reduced humoral immune responses to standard IIVs in immunosuppressed patients who had HIV, organ transplants, or cancer and those receiving immunosuppressive medications (*11*). The pooled odds of increased antibody titers after IIV ranged from 0.24 to 0.71 among immunosuppressive conditions compared with nonimmunosuppressive conditions (*11*). Studies of high-dose seasonal IIVs have demonstrated consistently stronger antibody responses (1.1–6.7-fold increase in antibody titers) compared with standard IIVs in adults <65 years of age (*18*,*19*,*36*,*38*,*39*). Studies of adjuvanted seasonal IIVs have not consistently resulted in improved immune responses compared with standard IIVs (*19*,*42*–*44*). Although these studies of high-dose IIVs offer hope for improving immune response in immunosuppressed patients, they are not likely to represent the entire spectrum of conditions that might affect the immune system and might not reflect actual clinical efficacy.

Our analysis offers a starting point for identifying patients that clinical trials of healthy participants typically do not capture and in whom protection from standard vaccines might be suboptimal. A great deal of heterogeneity exists in immunosuppressive conditions with varying degrees of immunosuppression and conditions that affect different components of the immune system. The conditions captured in our analysis probably represent the severe end of the immunosuppression spectrum. For example, we did not assess certain medical conditions associated with lesser degrees of immune suppression, such as diabetes and end-stage renal failure (*45*,*46*). The risk for disease is likely to vary among the immunosuppressive conditions identified in our study. However, from a public health perspective, a case definition of immunosuppression provides a target population for assessing overall risk for disease, rates of vaccination, and protective effects of vaccination.

The increased rate of ARI hospitalizations and influenza vaccination among immunosuppressed enrollees in MarketScan datasets suggests that these codes identified persons at increased risk for severe manifestations of infection. In addition to an increase in severe infections caused by immunosuppression, higher rates of ARI hospitalization might reflect differences in healthcare-seeking patterns or admission practices. Patients who have some immunosuppressive conditions also have had reduced immune responses to standard IIV (*11*) and thus might benefit from improved influenza vaccine strategies. Further research evaluating performance of influenza vaccines among the immunosuppressed cohort could help determine if expanded use of enhanced vaccines is warranted and cost-effective. The case definition for immunosuppressive conditions could also be used to evaluate influenza vaccine effectiveness in hospital-based observational studies or large administrative databases that individually link vaccination records to laboratory confirmed influenza (*12*,*47*). Last, evaluation of antibody and cellular immune responses to enhanced vaccines compared with standard vaccines in patients with these broad range of immunosuppressive conditions could help bridge the evidence gap that is needed to inform licensure and policy decisions for expanding the use of these vaccines.

Our results should be interpreted in the context of several limitations. We used a previously developed set of ICD codes for identifying active immunosuppression in patients who had sepsis, but not all patients with these conditions might have active immunosuppression. For example, although we specified codes that included HIV only when symptomatic, we cannot be certain about the degree of immunosuppression among patients who had ICD codes for symptomatic HIV/AIDS. Conversely, we might have missed other conditions that could be immunosuppressive. However, the approach proposed by Greenberg et al. is a reasonable start because these authors validated these codes of immunosuppression against medical records (sensitivity 87%, specificity 98%) and identified that these patients had higher risk for sepsis (*20*). However, this validation occurred at 1 hospital, and the accuracy of the codes might be affected by temporal differences in coding practices and among medical institutions.

In addition, the switch from ICD-9 to ICD-10 might have affected our case definition and needs further validation against individual medical records. We used a broad definition for ARI hospitalization, which is not specific to risk for influenza risk alone. We also did not expand the use of the ARI discharge codes to beyond the third position because it would reduce the positive predictive value of the code. A review demonstrated that ≈15% of winter ARI hospitalizations are attributable to influenza (*48*). However, the relative differences in respiratory diseases between potentially immunocompromised and nonimmunocompromised enrollees was informative and is consistent with the higher risk for severe complications from infectious illnesses, including influenza, in this population. MarketScan vaccination data probably underestimate true influenza vaccine coverage in the population, particularly for persons >65 years of age, because not all vaccinations are billed to insurance companies (*24*). However, the relative vaccination rates for immunosuppressed and nonimmunosuppressed persons were informative and unlikely to be affected.

Our study also considered an enrollee immunosuppressed during the study year if they met the case definition at any point in the year, but immunosuppression can be time-variant. Data from MarketScan represents a subset of the US insured population and might not be generalizable to other insured or noninsured populations (*49*,*50*). Claims-based data are also subject to inaccuracies and missingness. Insured patients are likely healthier than uninsured patients and thus our data may underestimate immunosuppression. Although we did not observe substantial increases in prevalence of immunosuppression, prevalence might be higher after the onset of the study period because of increasing coverage of these medications through insurance providers that are captured by MarketScan. Last, although some enrollees dropped out before the end of the 12-month study period, prevalence estimates would be unaffected if drop-out rates were similar between immunosuppressed and nonimmunosuppressed enrollees.

In conclusion, our findings quantify that immunosuppressive conditions, many of which impair immune responses to standard influenza vaccines, affect ≈6% of the enrollees in a large US claims database. Patients identified by our case definitions manifested higher risk for complications from respiratory infections, with 1 in 3 ARI hospitalizations occurring among patients who were immunosuppressed. Consequently, novel strategies to improve efficacy of influenza vaccines in these high-risk patients could substantially reduce the overall burden of severe influenza and hospitalizations in the population.
